# Enhancing Brain Connectivity With Infra-Low Frequency Neurofeedback During Aging: A Pilot Study

**DOI:** 10.3389/fnhum.2022.891547

**Published:** 2022-05-30

**Authors:** Olga R. Dobrushina, Larisa A. Dobrynina, Galina A. Arina, Elena I. Kremneva, Evgenia S. Novikova, Mariia V. Gubanova, Ekaterina V. Pechenkova, Anastasia D. Suslina, Vlada V. Aristova, Viktoriya V. Trubitsyna, Marina V. Krotenkova

**Affiliations:** ^1^Third Neurological Department, Research Center of Neurology, Moscow, Russia; ^2^Faculty of Psychology, M.V. Lomonosov Moscow State University, Moscow, Russia; ^3^Department of Radiology, Research Center of Neurology, Moscow, Russia; ^4^Laboratory for Cognitive Research, HSE University, Moscow, Russia

**Keywords:** endogenous neuromodulation, neurofeedback, infra-low frequency, aging, intrinsic connectivity networks, functional magnetic resonance imaging, resting state fMRI

## Abstract

Aging is associated with decreased functional connectivity in the main brain networks, which can underlie changes in cognitive and emotional processing. Neurofeedback is a promising non-pharmacological approach for the enhancement of brain connectivity. Previously, we showed that a single session of infra-low frequency neurofeedback results in increased connectivity between sensory processing networks in healthy young adults. In the current pilot study, we aimed to evaluate the possibility of enhancing brain connectivity during aging with the use of infra-low frequency neurofeedback. Nine females aged 52 ± 7 years with subclinical signs of emotional dysregulation, including anxiety, mild depression, and somatoform symptoms, underwent 15 sessions of training. A resting-state functional MRI scan was acquired before and after the training. A hypothesis-free intrinsic connectivity analysis showed increased connectivity in regions in the bilateral temporal fusiform cortex, right supplementary motor area, left amygdala, left temporal pole, and cerebellum. Next, a seed-to-voxel analysis for the revealed regions was performed using the post- vs. pre-neurofeedback contrast. Finally, to explore the whole network of neurofeedback-related connectivity changes, the regions revealed by the intrinsic connectivity and seed-to-voxel analyses were entered into a network-based statistical analysis. An extended network was revealed, including the temporal and occipital fusiform cortex, multiple areas from the visual cortex, the right posterior superior temporal sulcus, the amygdala, the temporal poles, the superior parietal lobule, and the supplementary motor cortex. Clinically, decreases in alexithymia, depression, and anxiety levels were observed. Thus, infra-low frequency neurofeedback appears to be a promising method for enhancing brain connectivity during aging, and subsequent sham-controlled studies utilizing larger samples are feasible.

## Introduction

Aging is widely known to be associated with cognitive decline, ranging from subclinical changes in memory and attention to severe dementia (Gauthier et al., 2006). Even mild cognitive impairment can lead to difficulties in activities with high cognitive demands and can be of high subjective significance for intellectually active people (Reppermund et al., 2013). An increasing number of studies show that aging also leads to changes in emotional processing. Late-life depression is common across populations (Luppa et al., 2012) and has a poorer prognosis compared to depression at younger ages (Mitchell and Subramaniam, 2005). Furthermore, older people show specific difficulties in understanding emotions (Phillips et al., 2002). According to the results of a large population-based study, aging is strongly associated with alexithymia—difficulties in the recognition and verbalization of emotions (Mattila et al., 2006). Importantly, both depression and alexithymia are independent cardiovascular risk factors, and cardiovascular diseases are a major cause of age-related mortality (Grabe et al., 2010; Tolmunen et al., 2010; Dhar and Barton, 2016). Thus, in addition to cognitive decline, the emotional decline may be a clinically important factor in aging.

The age-related emotional decline is thought to be based on altered functional activation and connectivity in the brain areas related to self-referential and socioemotional processing, including the visual and sensorimotor networks (Lyoo and Yoon, 2017) and the insular cortex (Dobrushina et al., 2020a). At the same time, emotional aging is associated with increased intra-network connectivity within the executive control network and also with increased inter-network connectivity between the executive control network and the default-mode network, suggesting a stronger top-down vs. bottom-up integration of self-referential information (Lyoo and Yoon, 2017). These data are in accordance with the posterior-anterior shift in aging (PASA) model, which proposes that age-related alterations in brain activation and connectivity mostly affect posterior regions, leading to a functional shift towards the frontal cortex (Davis et al., 2008; McCarthy et al., 2014).

Despite the general association of aging with cognitive and emotional declines, there is a high inter-individual variability, with some adults maintaining outstanding capabilities despite advancing age. The capacity for “brain maintenance” (Nyberg et al., 2012) may be partially explained by the adaptive functional reorganization of brain connectivity patterns (Sala-Llonch et al., 2015). Thus, it is of great significance to develop safe and practically advantageous methods for the enhancement of brain functional connectivity. Non-invasive neuromodulation refers to a group of non-pharmacological techniques for the improvement of brain functional states, and it has been shown to affect connectivity patterns (To et al., 2018). While most data come from studies on neurostimulation techniques such as transcranial magnetic stimulation and direct current stimulation, there is increasing evidence that neurofeedback can also be used to modulate brain intrinsic networks (Ros et al., 2013; Kluetsch et al., 2014; Emmert et al., 2016; Sitaram et al., 2017; Dobrushina et al., 2020b). Neurofeedback is less interventional than neurostimulation, since it is brain training rather than direct stimulation, and, in the form of electroencephalographic (EEG) neurofeedback, it can be delivered with the use of compact and affordable devices.

Currently, multiple EEG neurofeedback modalities are investigated and used in clinical practice, with the most popular approach being explicit up- or down-training of selected bandwidths (Omejc et al., 2019). Explicit trainings rely on a patient’s executive functioning and thus may be problematic to apply in individuals with cognitive decline and/or low motivation. In the current study, we investigate the feasibility of implicit infra-low frequency EEG neurofeedback in aging people. The infra-low frequency EEG domain (*f* < 0.1 Hz) is thought to be linked to the dynamics of intrinsic brain networks (Hiltunen et al., 2014; Haufe et al., 2018), and infra-low frequency neurofeedback is used to enhance emotional regulation and cognitive performance (Othmer, 2011; Othmer et al., 2013; Grin-Yatsenko et al., 2018). Implicit neurofeedback does not rely on executive functioning, which allows for the potential application of the method even in patients with profound cognitive impairment.

In a randomized sham-controlled study in healthy young adults, we previously showed that a single session of implicit infra-low frequency neurofeedback results in increased connectivity between the networks involved in multimodal sensory processing (Dobrushina et al., 2020b). Comparing the dynamics after real vs. sham neurofeedback, we revealed a network with increased post vs. pre connectivity, including the salience network regions (right anterior insula; left and right rostral prefrontal cortex), Wernicke’s and Broca’s areas in the language network, and the ventral visual pathway. As a follow-up step, we aimed to evaluate the long-term stability of the neurofeedback-related connectivity changes and also the transferability of the results to clinically significant groups. In the current pilot study, we tested the possibility of enhancing functional brain connectivity and improving emotional regulation in aging people with the use of infra-low frequency neurofeedback.

## Materials and Methods

### Participants and Experimental Design

Nine participants were enrolled in the intervention group from a sample of female non-physician healthcare workers who participated in our previous study on the role of emotional regulation and interoception in age-related white matter changes (Dobrushina et al., 2020a). The following inclusion criteria were used: age from 40 to 65 years; female; no history of cardiovascular events, such as stroke or myocardial infarction; no severe white matter hyperintensities according to structural MRI—modified Fazekas score of 2 or less (Fazekas et al., 1987); and the presence of subclinical signs of emotional dysregulation, including anxiety, mild depression, and somatoform symptoms. The participants reported no limitations in daily and professional activities. The study protocol was approved by the Ethics Committee and the Institutional Review Board of the Research Center of Neurology, and all participants gave informed consent for participation.

The intervention included 15 sessions of infra-low frequency neurofeedback (Othmer, 2017). At enrollment and after the course of neurofeedback the participants underwent resting-state functional magnetic resonance imaging (fMRI). The participants also completed a set of questionnaires: the Toronto Alexithymia Scale 20 (TAS-20, Bagby et al., 1994), the Beck Depression Inventory (BDI, Beck et al., 1961) and the Spielberger State-Trait Anxiety Inventory (STAI, Spielberger et al., 1983) a total of three times: 2 to 4 months before enrollment (as a part of the previous study; see Dobrushina et al., 2020a), at enrollment and after the neurofeedback course. The questionnaire data were analyzed in the R project (packages “ggpubr”, “ggplot2”, and “reshape”) with the use of the Friedman test with subsequent Wilcoxon pairwise comparisons (Bonferroni correction).

Results were verified with a control dataset of nine participants taken from a study by Schumann et al. (2021) (OpenNeuro Accession Number: ds003357^1^); the dataset contained resting-state fMRI data acquired before and after a course of heart-rate variability biofeedback training or control training. The sessions were performed five times a week during an 8-week study period, and each session included two training runs lasting for 11 min with a short pause in between. We selected nine older participants from the control group: six males and three females aged 34 ± 10 years (range 26−53 years). During the control training, the participants played jump and run video games with their heart-rate variability recorded in the background.

### Neurofeedback Procedure

Each participant from the intervention group received 15 sessions of infra-low frequency neurofeedback (Othmer, 2017) lasting 45 min and performed two to three times a week. EEG signals were recorded from Ag/AgCl sintered electrodes positioned at the P4 and T4 sites (10–20 system), while the reference electrode was placed at Cz and the ground electrode was placed on the forehead. The skin was prepared with NuPrep abrasive paste, and the electrodes were fixed with 10–20 conductive paste to reach an impedance below 5 kOhm. The EEG signal was recorded with a NeuroAmp (Corscience GmbH) DC-amplifier, sampled with 1 K samples per second, filtered and down-sampled to 250 samples per second, and 32-bit resolution. Cygnet biofeedback software (BEE Medic GmbH) was used for signal processing and feedback imagery presentation. In the Dreamscapes neurofeedback game (Somatic Vision Inc.), participants “moved” through an artificial landscape, with the speed of movement and brightness of the image governed in real time by the infra-low frequency band-limited waveform of the EEG signal. Due to the implicit principle of infra-low frequency neurofeedback, there was no goal to voluntarily influence the speed or brightness; the participants were instructed to observe the imagery, to “take a walk through the landscape.”

### fMRI Acquisition and Preprocessing

For the intervention group, MRI was performed with a Siemens MAGNETOM Verio 3T scanner (Erlangen, Germany) located at the Research Center of Neurology, Moscow. A three-dimensional structural image was acquired during the first scan (at enrollment), consisting of a sagittal T1-weighted 3D-MPRAGE sequence (TR 1,900 ms, TE 2.47 ms, voxel size 1 × 1 × 1 mm^3^, FOV 250 mm). Functional images were acquired twice (at enrollment and after the neurofeedback course) using EPI T2*-gradient echo imaging sequences (TR 2,400 ms, TE 30 ms, voxel size 3 × 3 × 3 mm^3^, FOV 192 mm). The eyes-closed resting-state sequence included 190 scans. Four extra functional volumes were acquired at the start of the session and discarded by the scanner software to prevent the usage of artifactual data obtained before the magnetic equilibrium could be reached. The magnetic field map further used for the correction of images was obtained with a double-echo gradient field map sequence.

The control dataset was acquired by Schumann et al. (2021) with the use of a Siemens Prisma 3T scanner. Eyes open resting state functional imaging was performed before and after the course of control trainings. During the session, 1,900 whole-brain volumes were acquired in about 15 min. T2*-weighted images were obtained using a multiband multislice GE-EPI sequence (TR 484 ms, TE 30 ms, FA 90°, multiband factor 8) with 56 contiguous transverse slices of 2.5 mm thickness. A series of high-resolution anatomical T1-weighted volume scans (MPRAGE) were obtained in a sagittal slice orientation (TR 2,300 ms, TE 3.03 ms, TI 900 ms, FA 9°, acquisition matrix 256 × 256 × 192, acceleration factor PAT 2) with an isotropic resolution of 1 mm^3^.

Pre-processing of the fMRI data was performed with the use of Statistical Parametric Mapping (SPM) 12^2^ (RRID:SCR_007037) and Conn 18b^3^ (RRID:SCR_009550) packages. The initial pre-processing in the SPM toolbox included slice-timing correction, calculation of the voxel displacement map, realignment and unwrapping of the functional images, co-registration of the structural images and the two sets of functional images, spatial normalization into standard Montreal Neurological Institute (MNI) space, segmentation of the anatomical image and spatial smoothing using a Gaussian kernel of 8 mm full width at half maximum.

Additional steps were performed in the Conn toolbox. Artifacts were addressed by the motion-scrubbing procedure (ART toolbox:^4^, RRID:SCR_005994), which involved the detection of outlier scans characterized by head displacement greater than 0.9 mm in any direction or deviation of the image global intensity by more than five standard deviations from the session mean; the outliers were further deweighted at the modeling stage. An anatomical component-based analysis of a CompCor method was applied to regress out the principal components of the BOLD-signal estimated from the white matter and cerebrospinal fluid volumes (Behzadi et al., 2007). Residual head motion parameters and their derivatives were regressed out from the signal. Temporal lowpass and highpass frequency filters were applied to the data, restricting the analysis to frequencies of 0.008–0.09 Hz.

### Functional Connectivity Analysis

The functional connectivity analysis of the intervention group data included three steps. First, we performed a hypothesis-free voxel-to-voxel intrinsic connectivity analysis (intrinsic connectivity contrast, ICC). ICC maps represent a measure of node centrality at each voxel, characterized by the strength of connectivity between a given voxel and the rest of the brain and defined as the root mean square of correlation coefficients between each individual voxel and all the voxels in the brain (Martuzzi et al., 2011). The ICC values entered the second-level general linear model, and group-level contrasts were obtained. Changes in the connectivity maps after the neurofeedback (post- vs. pre- sessions) were evaluated with a *T*-contrast (two-sided). Multiple comparison control was implemented with a false discovery error rate with *p* < 0.05 at the cluster-level, given a voxel-wise statistical threshold of *p* < 0.001 uncorrected.

Second, the clusters revealed by the ICC analysis (six clusters) were entered as regions of interest (ROI) in a seed-to-voxel connectivity analysis. Changes in the connectivity maps for each ROI after the neurofeedback (post- vs. pre- sessions) were evaluated with a *T*-contrast (two-sided). Multiple comparison control was implemented with a false discovery error rate with *p* < 0.05 at the cluster-level, with additional Bonferroni correction for the number of ROIs (*p* = 0.05/6 = 0.008), given a voxel-wise statistical threshold of *p* < 0.001 uncorrected.

Third, to reveal the network of connectivity changes associated with the neurofeedback, the clusters revealed by both the ICC and seed-to-voxel analyses were entered into an ROI-to-ROI analysis (a total of 16 ROI). Overlapping clusters were joined with the use of MarsBaR toolbox^5^ (RRID:SCR_009605). A network-based statistics analysis by intensity was applied for the post- vs. pre-condition with a false discovery error rate (*p* < 0.001), given a connection threshold of *p* < 0.01 uncorrected. Network-based statistics allowed us to test hypotheses about interconnected sets, or clusters of connections (networks) rather than individual connections (Zalesky et al., 2010).

For the control dataset, we followed the same analytic strategy. First, we performed an ICC analysis for the post- vs. pre- sessions. Second, we entered the single cluster revealed by ICC as an ROI into the seed-to-voxel analysis. Third, we performed a network-based ROI-to-ROI analysis for the same set of ROIs as in the intervention group (16 ROIs) to search for possible changes in this contour after the control intervention (post- vs. pre- sessions). For the control dataset, we used the same statistical methods and thresholds as in the intervention group.

## Results

### Voxel-to-Voxel Intrinsic Connectivity Analysis

**Figure 1:**
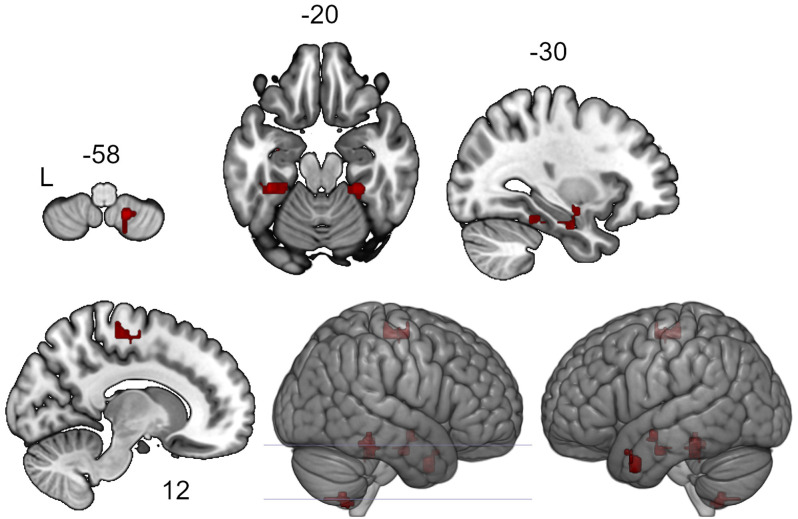
Clusters showing increased intrinsic connectivity after neurofeedback. The connectivity map for intrinsic connectivity contrast post- vs. pre- neurofeedback is thresholded at *p*-FDR < 0.05 at the cluster-level with a cluster-defining voxel-wise statistical threshold of *p* < 0.001 uncorrected.

According to the results of the ICC analysis, clusters in the left and right temporal fusiform cortex, the right supplementary motor area, the left amygdala, the left temporal pole and the cerebellum showed increased functional connectivity after the neurofeedback (see Figure 1, Table 1). The six clusters were entered as ROIs in the subsequent seed-to-voxel connectivity analysis.

**Table 1:** Clusters showing increased intrinsic connectivity after neurofeedback.

Cluster localization	MNI coordinates	Cluster size,	Cluster size *p*-FDR
	(x, y, z) of the peak	voxels (3 × 3 × 3 mm^3^)	
Left temporal fusiform cortex, posterior division	−18 −34 −28	136	0.0002
Right supplementary motor area	+16 −10 +58	113	0.0005
Right temporal fusiform cortex, posterior division	+28 −38 −22	102	0.0008
Left amygdala	−32 −08 −14	83	0.002
Left temporal pole	−50 +04 −32	82	0.002
Right cerebellum	+16 −56 −58	64	0.007

In the control dataset, the ICC analysis revealed a single cluster in the right posterior superior temporal gyrus, homologous to Wernicke’s area (peak +70 −28 +10, 42 voxels with voxel size 2.5 × 2.5 × 2.5 mm^3^, cluster size *p*-FDR < 0.01), with increased connectivity after the course of training.

### Seed-to-Voxel Analysis

**Figure 2:**
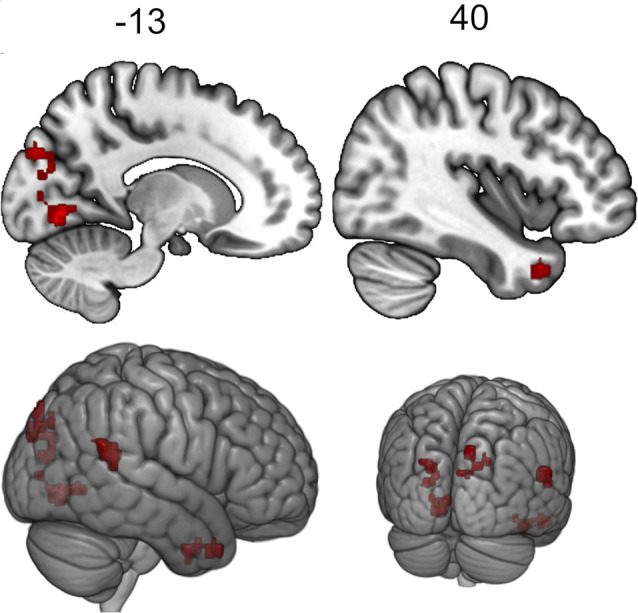
Clusters showing increased connectivity with the left posterior temporal fusiform cortex after neurofeedback. The connectivity map for intrinsic connectivity contrast post- vs.pre-neurofeedback is thresholded at *p*-FDR < 0.008 at the cluster-level with a cluster-defining voxel-wise statistical threshold of *p* < 0.001 uncorrected.

**Figure 3:**
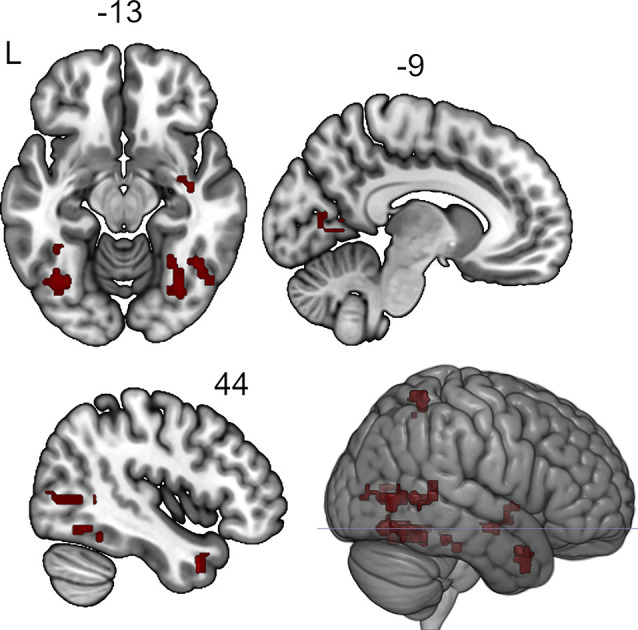
Clusters showing increased connectivity with the right posterior temporal fusiform cortex after neurofeedback. The connectivity map for intrinsic connectivity contrast post- vs.pre-neurofeedback is thresholded at *p*-FDR < 0.008 at the cluster-level with a cluster-defining voxel-wise statistical threshold of *p* < 0.001 uncorrected.

**Figure 4:**
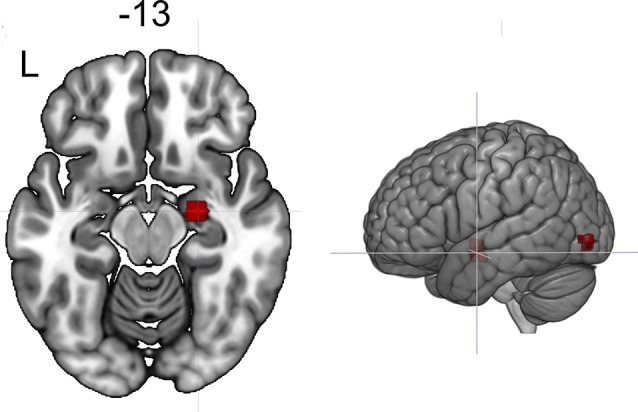
Clusters showing increased connectivity with the left amygdala after neurofeedback. The connectivity map for intrinsic connectivity contrast post- vs. pre-neurofeedback is thresholded at *p*-FDR < 0.008 at the cluster-level with a cluster-defining voxel-wise statistical threshold of *p* < 0.001 uncorrected.

The seed-to-voxel analysis for the neurofeedback group revealed increased post- vs. pre- connectivity for the seed ROIs in the left and right posterior temporal fusiform cortex and in the left amygdala (Table 2). The revealed areas were mainly located in the lateral and medial occipital cortex, as well as in the right temporal pole and amygdala (Figures 2, 3, 4).

**Table 2:** Clusters showing increased connectivity with the seed regions after neurofeedback.

Seed ROI	Localization of revealed clusters	MNI coordinates (x, y, z) of the peak	Cluster size, voxels (3 × 3 × 3 mm^3^)	Cluster size *p*-FDR
Left temporal fusiform cortex, posterior division (−18 −34 −28)	Left lingual gurus	−12 −74 −04	182	0.00007
	Right posterior superior temporal sulcus	+60 −50 +22	177	0.00007
	Left lateral occipital cortex, superior division, extending to cuneus	−14 −88 +32	161	0.0001
	Right temporal pole	+40 +14 −34	148	0.0001
	Right lateral occipital cortex, superior division	+10 −92 +28	118	0.0006
Right temporal fusiform cortex, posterior division (+28 −38 −22)	Right inferior lateral occipital cortex, middle temporal gurus, posterior superior temporal sulcus	+52 −68 +02	335	<0.000001
	Right occipital fusiform cortex	+34 −70 −10	272	0.000002
	Left occipital fusiform cortex	−38 −70 −10	266	0.000002
	Right superior parietal lobule	+30 −52 +64	104	0.002
	Left intracalcarine cortex	−06 −64 +02	104	0.002
	Right temporal pole	+48 +10 −28	99	0.003
Left amygdala (−32 −08 −14)	Right amygdala	+30 −10 −08	155	0.0007
	Left lateral occipital cortex, inferior division	−42 −86 −04	128	0.001

For the control dataset, the seed-to-voxel analysis revealed increased post- vs. pre-connectivity of the ROI in the right posterior superior temporal gyrus with areas from the sensorimotor and visual cortex (Table 3).

**Table 3:** Clusters showing increased connectivity with the seed regions after the control trainings.

Seed ROI	Localization of revealed clusters	MNI coordinates (x, y, z) of the peak	Cluster size, voxels (2.5 × 2.5 × 2.5 mm^3^)	Cluster size *p*-FDR
Right posterior superior temporal gyrus (+70 −28 +10)	Left postcentral gyrus	−50 −04 +34	173	<0.0000001
	Left lateral occipital cortex, superior division	−18 −64 +56	109	0.000008
	Right postcentral gyrus	+42 −32 +50	109	0.000008
	Left cuneal cortex	−14 −80 +32	90	0.00003
	Right precentral gyrus	+52 −02 +28	59	0.0004

### Network-Based Statistics Analysis

To explore the network, related to the effects of neurofeedback, we entered the ROI revealed both in the ICC and seed-to-voxel analyses (Tables s 1, 2) into an ROI-to-ROI analysis. The following overlapping clusters were joined pairwise: the right inferior lateral occipital cortex and the posterior superior temporal sulcus; the left lingual gyrus and the intracalcarine cortex; and the right temporal pole (seed-to-voxel results from the left and right temporal fusiform cortex). Thus, 16 ROIs entered the analysis.

**Figure 5:**
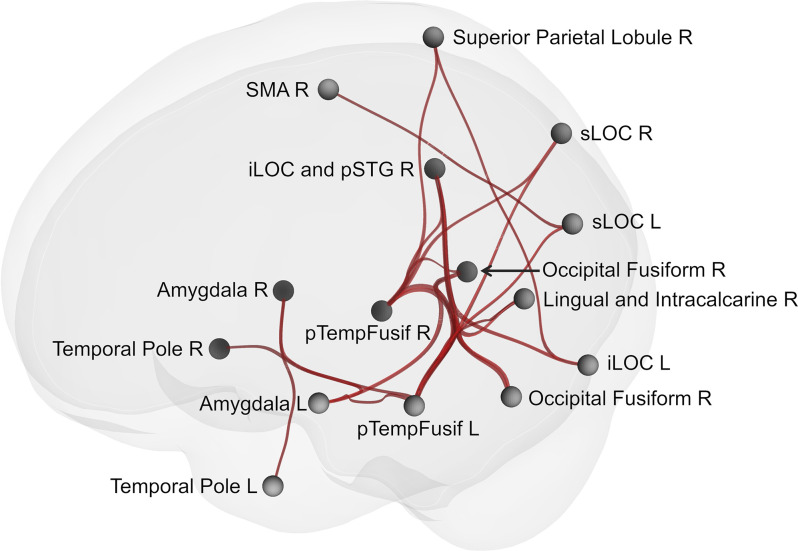
Brain network showing increased connectivity after the course of infra-low frequency neurofeedback. The network is thresholded for the post- vs. pre- neurofeedback condition with a false discovery error rate of *p* < 0.001 by intensity, given a connection threshold of *p* < 0.01 uncorrected. iLOC, inferior lateral occipital cortex; pTempFusiform, posterior temporal fusiform cortex; sLOC, superior lateral occipital cortex; SMA, supplementary motor area; SMG, supramarginal gyrus.

**Figure 6:**
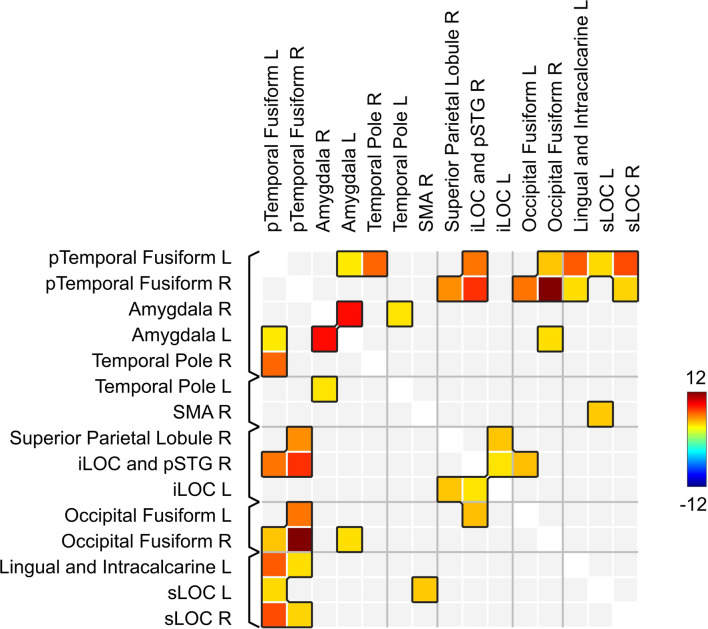
Matrix of connections within the network related to the effects of infra-low frequency neurofeedback. iLOC, inferior lateral occipital cortex; pTemporal, posterior temporal; sLOC, superior lateral occipital cortex; SMA, supplementary motor area; pSTS, posterior superior temporal sulcus.

Network-based statistics analysis for the neurofeedback group revealed a network including 15 positively connected ROIs (*p*-FDR < 0.0000001; all except the right cerebellum), with the main hubs represented by the left and right posterior temporal fusiform cortex (see Figures 5, 6).

The network-based analysis for the control dataset revealed no results, even at lowered statistical thresholds (*p*-FDR < 0.05 NBS by intensity, given a connection threshold of *p* < 0.05 uncorrected).

### Dynamics of Emotional Regulation

**Figure 7:**
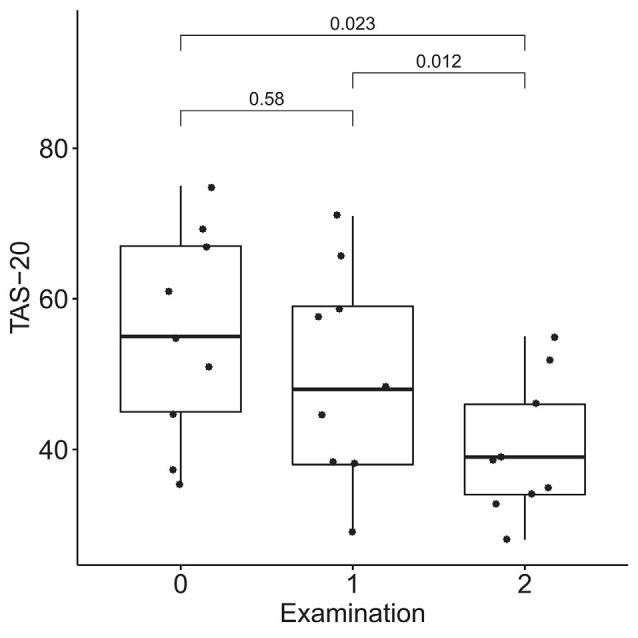
Dynamics of alexithymia after the course of infra-low frequency neurofeedback. The examination was performed three times: (0) 2 to 4 months before enrollment, (1) at enrollment; and (2) after the neurofeedback course. Significance for the Wilcoxon pairwise comparisons is presented on the plot (*p*-value, Bonferroni corrected).

**Figure 8:**
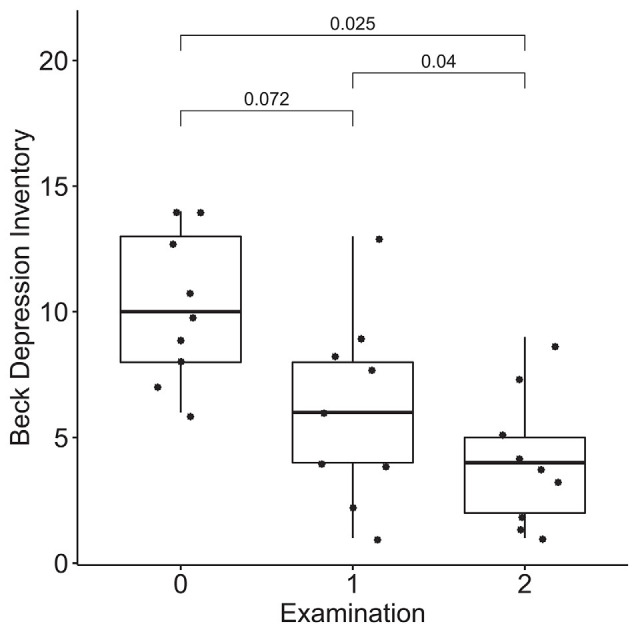
Dynamics of depression after the course of infra-low frequency neurofeedback. The examination was performed three times: (0) 2 to 4 months before enrollment, (1) at enrollment; and (2) after the neurofeedback course. Significance for the Wilcoxon pairwise comparisons is presented on the plot (*p*-value, Bonferroni corrected).

**Figure 9:**
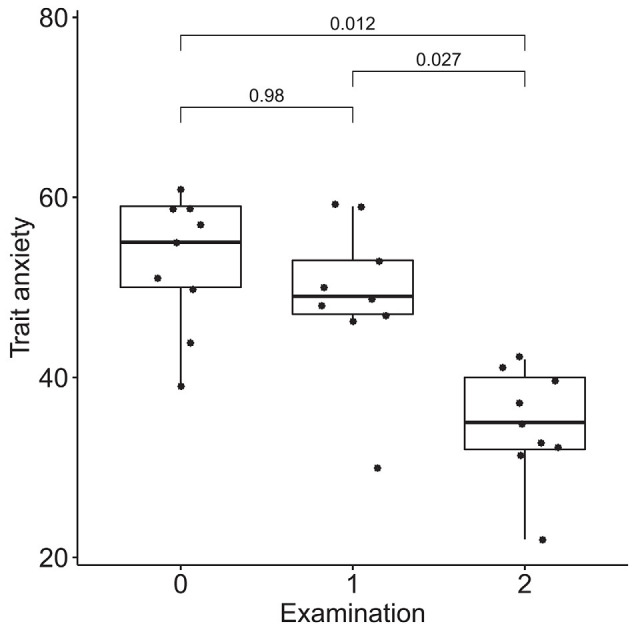
Dynamics of trait anxiety after the course of infra-low frequency neurofeedback. The examination was performed three times: (0) 2 to 4 months before enrollment, (1) at enrollment; and (2) after the neurofeedback course. Significance for the Wilcoxon pairwise comparisons is presented on the plot (*p*-value, Bonferroni corrected).

**Figure 10:**
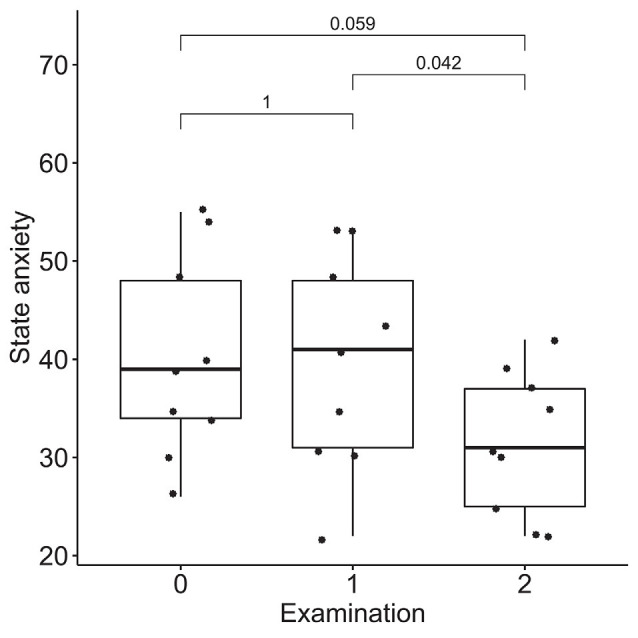
Dynamics of state anxiety after the course of infra-low frequency neurofeedback. The examination was performed three times: (0) 2 to 4 months before enrollment, (1) at enrollment; and (2) after the neurofeedback course. Significance for the Wilcoxon pairwise comparisons is presented on the plot (*p*-value, Bonferroni corrected).

According to the results of the Friedman test, significant dynamics were observed for alexithymia measured with the TAS-20 (means of 55 ± 14, 50 ± 14, and 40 ± 9 for the three examinations; *p* = 0.004), depression measured with BDI (10 ± 3, 6 ± 4 and 4 ± 3 for the three examinations; *p* = 0.0004), STAI trait anxiety (53 ± 7, 49 ± 9 and 35 ± 6 for the three examinations; *p* = 0.0009) and state anxiety (40 ± 10, 40 ± 11, 31 ± 7 for the three examinations; *p* = 0.027). Pairwise comparisons revealed the difference between the examinations before and after the neurofeedback course, but not before and after the pre-study waiting period (Figures 7–10). Thus, the observed improvement likely developed due to the intervention rather than spontaneously.

## Discussion

In the current study, we observed strong changes in brain connectivity after the course of infra-low frequency neurofeedback. An extended network including the temporal and occipital fusiform cortex, multiple areas from the visual cortex (lateral occipital cortex, lingual and intracalcarine cortex), the right posterior superior temporal sulcus, the amygdala, the temporal poles, the superior parietal lobule, and the supplementary motor cortex showed increased connectivity post- vs. pre-neurofeedback. The fusiform cortex, which formed the main hubs of the revealed network, is involved in high-level visual processing tasks, such as the processing of human faces and bodies (Peelen and Downing, 2005; Rangarajan et al., 2014), and it is known to be anatomically and functionally connected to the lingual gyrus and the lateral occipital cortex (Rokem et al., 2017; Palejwala et al., 2020). The right posterior superior temporal sulcus, the amygdala, and the temporal poles also participate in facial perception (Gobbini and Haxby, 2007; Rokem et al., 2017), as well as in social cognition in general (Schurz et al., 2014). Specifically, the amygdala, a part of the salience network, is involved in the evaluation of face typicality (Todorov, 2012), and the temporal poles, which are a part of the default-mode network, are involved in the recruitment of biographical memories (Gobbini and Haxby, 2007; Li et al., 2014). The superior parietal lobule is mainly associated with visuospatial perception and attention, including the representation and manipulation of objects (Binkofski et al., 1999), while the supplementary motor area is involved in the execution of complex movements, including motor imagery (Szameitat et al., 2007; Nachev et al., 2008).

Taking together, the revealed network may be responsible for the processing of social stimuli, such as human faces, including the evaluation of their subjective salience in the biographical context (amygdala and temporal poles) and imagination of social interactions (supplementary motor area and superior parietal lobule). The changes in connectivity observed after neurofeedback go in the opposite direction of the age-related changes in brain connectivity of the networks involved in socioemotional processing. Contrary to the described age-related posterior-anterior shift (Davis et al., 2008; McCarthy et al., 2014; Lyoo and Yoon, 2017), we observed enhanced connectivity in the posterior part of the brain. Thus, the results of our study indicate the possibility of the therapeutic modulation of brain intrinsic networks during aging with infra-low frequency neurofeedback. Neurophysiological changes are shown to have a prognostic value during aging: networks disruption is a characteristic of progressive vs. stable mild cognitive impairment (Pusil et al., 2019). Further research is necessary to evaluate the therapeutic significance of interventions aimed at the enhancement of brain connectivity.

In our previous study on the immediate connectivity changes after a single session of infra-low frequency neurofeedback, we revealed a network with a different location, which, nevertheless, may be linked to the findings of the current study by its functional meaning (Dobrushina et al., 2020b). A single neurofeedback session resulted in the development of a multisensory network including areas from the speech, visual, and salience networks. Multisensory processing underlies the ability to perceive emotions and to give subjective meaning to social situations (Barrett, 2017; Critchley and Garfinkel, 2017). Long-term indirect stimulation of multisensory processing with infra-low frequency neurofeedback during a course of neurofeedback might have resulted in an enhanced functioning of the brain networks related to social perception—the changes we observed in the current study. Besides the assessment of long-lasting vs. immediate effects, our two studies differ in the age groups of the participants (older vs. young and middle-aged adults), and this factor might also contribute to the difference in findings.

The changes in brain connectivity were accompanied by a decrease in alexithymia, depression, and anxiety scores. Participants in our study demonstrated elevated baseline levels of alexithymia, consistent with the known age-related increase in alexithymia rates (Mattila et al., 2006). Despite alexithymia often being viewed as a trait, multiple studies show the state-like characteristics of alexithymia, with a possibility to achieve a decrease in alexithymia with psychotherapeutic interventions such as emotional intelligence training and mindfulness (Amani et al., 2013; Norman et al., 2019). On the basis of our preliminary results, it can be suggested that infra-low frequency neurofeedback also has therapeutic potential in individuals with alexithymia. A decrease in alexithymia is consistent with the observed increased connectivity in the network involved in the processing of social stimuli.

Alexithymia is strongly associated with depression, in older adults (Honkalampi et al., 2000b; Bamonti et al., 2010) and individuals with anxiety disorders (Berardis et al., 2008). It is suggested that ineffective emotional regulation may compromise the ability to manage social stressors in alexithymic individuals, making them more susceptible to affective disorders; this is known as the vulnerability hypothesis (Hemming et al., 2019). Thus, the observed concomitant decreases in alexithymia, depression, and anxiety after neurofeedback might be due to improved emotional regulation. At the same time, we cannot exclude the possibility that, conversely, a decrease in anxiety and depression levels resulted in secondary improved alexithymia scores, in accordance with the reactivity hypothesis of the association between the alexithymia and affective disorders (Honkalampi et al., 2000a).

In the control dataset taken from the study by Schumann et al. (2021), we observed no changes in the neural contour revealed in the neurofeedback group. Instead, increased connectivity of the right posterior superior temporal gyrus with areas from the sensorimotor cortex and visual cortex was evident after the course of trainings. These changes can be related to the training effect of the jump and run videogames that were used as a control intervention. Besides having a supplementary role in language processing, the right posterior superior temporal gyrus is known to participate in audiovisual integration (Specht and Wigglesworth, 2018), and the jump and run games were accompanied by sounds indicating jumps and steps. This explanation is in accordance with the observed connectivity with the sensorimotor cortex (running movement) and the visual cortex (processing of the video image). Thus, we propose that the effects observed in the neurofeedback group cannot be related to repeated scanning, as no common changes were found in the two datasets. The changes were specific and may be attributed to the participants’ experience in each study (i.e., the neurofeedback procedure vs. control video games).

Our study is a pilot investigation of the therapeutic potential of infra-low frequency neurofeedback in aging adults; it has important limitations. First, we included a small number of participants. Second, the study was not a randomized sham-controlled trial. For the neuroimaging part of the study, we used a control dataset acquired by other researchers with a different control intervention and a different resting-state fMRI scanning sequence. For the behavioral data, we used a pre-study waiting period to control for the possible spontaneous changes in alexithymia, depression, and anxiety. Third, in the current study, we focused on the emotional rather than the cognitive consequences of aging, so we cannot make inferences regarding the cognitive effects of the neurofeedback intervention and the possible mediating role of cognitive processes in emotional regulation dynamics. Fourth, the study included participants aged 52 ± 7 years, since we supposed that early age-related changes may be more sensible for therapeutic modulation; the potential effects of neurofeedback in older adults remain to be investigated. The results of our preliminary study cannot be used to guide clinical decisions. However, they illustrate the feasibility of endogenous neuromodulation in age-related brain changes, highlight the need for further research and provide the information necessary for designing subsequent randomized sham-controlled studies.

## Data Availability Statement

The datasets presented in this study can be found in online repositories. The names of the repository/repositories and accession number(s) can be found below: OpenNeuro repository, reference number ds004101. URL: https://doi.org/10.18112/openneuro.ds004101.v1.0.1.

## Ethics Statement

The studies involving human participants were reviewed and approved by Ethics Committee and the Institutional Review Board of the Research Center of Neurology. The patients/participants provided their written informed consent to participate in this study.

## Author Contributions

OD, LD, GA, EK, and MK designed and coordinated the study. EN, MG, AS, VA, and VT collected the data. OD and EP performed the data analysis. All authors contributed to the article and approved the submitted version.

## Conflict of Interest

The authors declare that the research was conducted in the absence of any commercial or financial relationships that could be construed as a potential conflict of interest.

## Publisher’s Note

All claims expressed in this article are solely those of the authors and do not necessarily represent those of their affiliated organizations, or those of the publisher, the editors and the reviewers. Any product that may be evaluated in this article, or claim that may be made by its manufacturer, is not guaranteed or endorsed by the publisher.

## Footnotes

^1^ https://doi.org/10.1101/2020.08.10.243998

^2^ https://www.fil.ion.ucl.ac.uk/spm

^3^ http://www.nitrc.org/projects/conn

^4^ http://www.nitrc.org/projects/artifact_detect

^5^ https://marsbar-toolbox.github.io

## References

[B1] Amani M. Goodarzi M. Ahamadian H. (2013). Efficiency of training emotional intelligence on reducing alexithymia syndrome in third grade male high school students. Int. Lett. Soc. Hum. Sci. 12, 7–13. 10.18052/www.scipress.com/ILSHS.12.7

[B2] Bagby R. M. Parker J. D. A. Taylor G. J. (1994). The twenty-item Toronto Alexithymia scale—I. Item selection and cross-validation of the factor structure. J. Psychosom. Res. 38, 23–32. 10.1016/0022-3999(94)90005-1 8126686

[B3] Bamonti P. M. Heisel M. J. Topciu R. A. Franus N. Talbot N. L. Duberstein P. R. (2010). Association of alexithymia and depression symptom severity in adults aged 50 years and older. Am. J. Geriatr. Psychiatry 18, 51–56. 10.1097/JGP.0b013e3181bd1bfe 20094018 PMC3071987

[B4] Barrett L. F. (2017). The theory of constructed emotion: an active inference account of interoception and categorization. Soc. Cogn. Affect Neurosci. 12, 1–23. 10.1093/scan/nsw154 27798257 PMC5390700

[B5] Beck A. T. Ward C. H. Mendelson M. Mock J. Erbaugh J. (1961). An inventory for measuring depression. Arch. Gen. Psychiatry 4, 561–571. 10.1001/archpsyc.1961.01710120031004 13688369

[B102] Behzadi Y. Restom K. Liau J. Liu T. T. (2007). A component based noise correction method (CompCor) for BOLD and perfusion based fMRI. Neuroimage 37, 90–101. 10.1016/j.neuroimage.2007.04.042 17560126 PMC2214855

[B6] Berardis D. Campanella D. Nicola S. Gianna S. Alessandro C. Chiara C.. (2008). The impact of alexithymia on anxiety disorders: a review of the literature. Curr. Psychiatry Rev. 4, 80–86. 10.2174/157340008784529287

[B7] Binkofski F. Buccino G. Posse S. Seitz R. J. Rizzolatti G. Freund H.-J. (1999). A fronto-parietal circuit for object manipulation in man: evidence from an fMRI-study. Eur. J. Neurosci. 11, 3276–3286. 10.1046/j.1460-9568.1999.00753.x 10510191

[B8] Critchley H. D. Garfinkel S. N. (2017). Interoception and emotion. Curr. Opin. Psychol. 17, 7–14. 10.1016/j.copsyc.2017.04.020 28950976

[B9] Davis S. W. Dennis N. A. Daselaar S. M. Fleck M. S. Cabeza R. (2008). Que PASA? The posterior-anterior shift in aging. Cereb. Cortex 18, 1201–1209. 10.1093/cercor/bhm155 17925295 PMC2760260

[B10] Dhar A. K. Barton D. A. (2016). Depression and the link with cardiovascular disease. Front. Psychiatry 7:11. 10.3389/fpsyt.2016.00033 27047396 PMC4800172

[B11] Dobrushina O. R. Arina G. A. Dobrynina L. A. Suslina A. D. Solodchik P. O. Belopasova A. V.. (2020a). The ability to understand emotions is associated with interoception-related insular activation and white matter integrity during aging. Psychophysiology 57:e13537. 10.1111/psyp.13537 31994733

[B12] Dobrushina O. R. Vlasova R. M. Rumshiskaya A. D. Litvinova L. D. Mershina E. A. Sinitsyn V. E.. (2020b). Modulation of intrinsic brain connectivity by implicit electroencephalographic neurofeedback. Front. Hum. Neurosci. 14:192. 10.3389/fnhum.2020.00192 32655386 PMC7324903

[B13] Emmert K. Kopel R. Sulzer J. Brühl A. B. Berman B. D. Linden D. E. J.. (2016). Meta-analysis of real-time fMRI neurofeedback studies using individual participant data: how is brain regulation mediated. Neuroimage 124, 806–812. 10.1016/j.neuroimage.2015.09.042 26419389

[B14] Fazekas F. Chawluk J. Alavi A. Hurtig H. Zimmerman R. (1987). MR signal abnormalities at 1.5 T in Alzheimer’s dementia and normal aging. Am. J. Roentgenol. 149, 351–356. 10.2214/ajr.149.2.351 3496763

[B15] Gauthier S. Reisberg B. Zaudig M. Petersen R. C. Ritchie K. Broich K.. (2006). Mild cognitive impairment. Lancet 367, 1262–1270. 10.1016/S0140-6736(06)68542-5 16631882

[B16] Gobbini M. I. Haxby J. v. (2007). Neural systems for recognition of familiar faces. Neuropsychologia 45, 32–41. 10.1016/j.neuropsychologia.2006.04.015 16797608

[B17] Grabe H. J. Schwahn C. Barnow S. Spitzer C. John U. Freyberger H. J.. (2010). Alexithymia, hypertension and subclinical atherosclerosis in the general population. J. Psychosom. Res. 68, 139–147. 10.1016/j.jpsychores.2009.07.015 20105696

[B18] Grin-Yatsenko V. Othmer S. Ponomarev V. Evdokimov S. Konoplev Y. Kropotov J. (2018). Infra-low frequency neurofeedback in depression: three case studies. NeuroRegulation 5, 30–42. 10.15540/nr.5.1.30

[B100] Haufe S. DeGuzman P. Henin S. Arcaro M. Honey C. J. Hasson U.. (2018). Elucidating relations between fMRI, ECoG, and EEG through a common natural stimulus. Neuroimage 179, 79–91. 10.1016/j.neuroimage.2018.06.016 29902585 PMC6063527

[B19] Hemming L. Haddock G. Shaw J. Pratt D. (2019). Alexithymia and its associations with depression, suicidality and aggression: an overview of the literature. Front. Psychiatry 10:203. 10.3389/fpsyt.2019.00203 31031655 PMC6470633

[B101] Hiltunen T. Kantola J. Abou Elseoud A. Lepola P. Suominen K. Starck T.. (2014). Infra-slow EEG fluctuations are correlated with resting-state network dynamics in fMRI. J. Neurosci. 34, 356–362. 10.1523/JNEUROSCI.0276-13.2014 24403137 PMC6608153

[B20] Honkalampi K. Hintikka J. Saarinen P. Lehtonen J. Viinamäki H. (2000a). Is alexithymia a permanent feature in depressed patients? Results from a 6-month follow-up study. Psychother. Psychosom. 69, 303–308. 10.1159/000012412 11070442

[B21] Honkalampi K. Hintikka J. Tanskanen A. Lehtonen J. Viinamäki H. (2000b). Depression is strongly associated with alexithymia in the general population. J. Psychosom. Res. 48, 99–104. 10.1016/s0022-3999(99)00083-5 10750635

[B22] Kluetsch R. C. Ros T. Théberge J. Frewen P. A. Calhoun V. D. Schmahl C.. (2014). Plastic modulation of PTSD resting-state networks and subjective wellbeing by EEG neurofeedback. Acta Psychiatr. Scand. 130, 123–136. 10.1111/acps.12229 24266644 PMC4442612

[B23] Li W. Mai X. Liu C. (2014). The default mode network and social understanding of others: what do brain connectivity studies tell us. Front. Hum. Neurosci. 8:74. 10.3389/fnhum.2014.00074 24605094 PMC3932552

[B24] Luppa M. Sikorski C. Luck T. Ehreke L. Konnopka A. Wiese B.. (2012). Age- and gender-specific prevalence of depression in latest-life - systematic review and meta-analysis. J. Affect. Disord. 136, 212–221. 10.1016/j.jad.2010.11.033 21194754

[B25] Lyoo Y. Yoon S. (2017). Brain network correlates of emotional aging. Sci. Rep. 7:15576. 10.1038/s41598-017-15572-6 29138429 PMC5686193

[B26] Martuzzi R. Ramani R. Qiu M. Shen X. Papademetris X. Constable R. T. (2011). A whole-brain voxel based measure of intrinsic connectivity contrast reveals local changes in tissue connectivity with anesthetic without a priori assumptions on thresholds or regions of interest. Neuroimage 58, 1044–1050. 10.1016/j.neuroimage.2011.06.075 21763437 PMC3183817

[B27] Mattila A. K. Salminen J. K. Nummi T. Joukamaa M. (2006). Age is strongly associated with alexithymia in the general population. J. Psychosom. Res. 61, 629–635. 10.1016/j.jpsychores.2006.04.013 17084140

[B28] McCarthy P. Benuskova L. Franz E. A. (2014). The age-related posterior-anterior shift as revealed by voxelwise analysis of functional brain networks. Front. Aging Neurosci. 6:301. 10.3389/fnagi.2014.00301 25426065 PMC4224138

[B29] Mitchell A. J. Subramaniam H. (2005). Prognosis of depression in old age compared to middle age: a systematic review of comparative studies. Am. J. Psychiatry 162, 1588–1601. 10.1176/appi.ajp.162.9.1588 16135616

[B30] Nachev P. Kennard C. Husain M. (2008). Functional role of the supplementary and pre-supplementary motor areas. Nat. Rev. Neurosci. 9, 856–869. 10.1038/nrn2478 18843271

[B31] Norman H. Marzano L. Coulson M. Oskis A. (2019). Effects of mindfulness-based interventions on alexithymia: a systematic review. Evid. Based Ment. Health 22, 36–43. 10.1136/ebmental-2018-300029 30077988 PMC10270453

[B32] Nyberg L. Lövdén M. Riklund K. Lindenberger U. Bäckman L. (2012). Memory aging and brain maintenance. Trends Cogn. Sci. 16, 292–305. 10.1016/j.tics.2012.04.005 22542563

[B33] Omejc N. Rojc B. Battaglini P. P. Marusic U. (2019). Review of the therapeutic neurofeedback method using electroencephalography: EEG neurofeedback. Bosn. J. Basic Med. Sci. 19:213. 10.17305/bjbms.2018.3785 30465705 PMC6716090

[B34] Othmer S. (2011). Psychological Health and Neurofeedback: Remediating PTSD and TBI. Los Angeles, CA: EEG Info. Available online at: http://www.misb.dk/artikler/NF_ved_PTSD.pdf.

[B35] Othmer S. F. (2017). Protocol Guide for Neurofeedback Clinicians, 5th Edn. Los Angeles, CA: EEG Info.

[B36] Othmer S. Othmer S. F. Kaiser D. A. Putman J. (2013). Endogenous neuromodulation at infralow frequencies. Semin Pediatr. Neurol. 20, 246–257. 10.1016/j.spen.2013.10.006 24365573

[B37] Palejwala A. H. O’Connor K. P. Milton C. K. Anderson C. Pelargos P. Briggs R. G.. (2020). Anatomy and white matter connections of the fusiform gyrus. Sci. Rep. 10:13489. 10.1038/s41598-020-70410-6 32778667 PMC7417738

[B38] Peelen M. v. Downing P. E. (2005). Selectivity for the human body in the fusiform gyrus. J. Neurophysiol. 93, 603–608. 10.1152/jn.00513.2004 15295012

[B39] Phillips L. H. MacLean R. D. J. Allen R. (2002). Age and the understanding of emotions: neuropsychological and sociocognitive perspectives. J. Gerontol B Psychol. Sci. Soc. Sci. 57, P526–P530. 10.1093/geronb/57.6.p526 12426435

[B40] Pusil S. López M. E. Cuesta P. Bruña R. Pereda E. Maestú F. (2019). Hypersynchronization in mild cognitive impairment: the “X” model. Brain 142, 3936–3950. 10.1093/brain/awz320 31633176

[B41] Rangarajan V. Hermes D. Foster B. L. Weiner K. S. Jacques C. Grill-Spector K.. (2014). Electrical stimulation of the left and right human fusiform gyrus causes different effects in conscious face perception. J. Neurosci. 34, 12828–12836. 10.1523/JNEUROSCI.0527-14.2014 25232118 PMC4166163

[B42] Reppermund S. Brodaty H. Crawford J. D. Kochan N. A. Draper B. Slavin M. J.. (2013). Impairment in instrumental activities of daily living with high cognitive demand is an early marker of mild cognitive impairment: the Sydney Memory and Ageing Study. Psychol. Med. 43, 2437–2445. 10.1017/S003329171200308X 23308393

[B43] Rokem A. Takemura H. Bock A. S. Scherf K. S. Behrmann M. Wandell B. A.. (2017). The visual white matter: the application of diffusion MRI and fiber tractography to vision science. J. Vis. 17:4. 10.1167/17.2.4 28196374 PMC5317208

[B44] Ros T. Théberge J. Frewen P. A. Kluetsch R. Densmore M. Calhoun V. D.. (2013). Mind over chatter: plastic up-regulation of the fMRI salience network directly after EEG neurofeedback. Neuroimage 65, 324–335. 10.1016/j.neuroimage.2012.09.046 23022326 PMC5051955

[B45] Sala-Llonch R. Bartres-Faz D. Junque C. (2015). Reorganization of brain networks in aging: a review of functional connectivity studies. Front. Psychol. 6:663. 10.3389/fpsyg.2015.00663 26052298 PMC4439539

[B46] Schumann A. de la Cruz F. Köhler S. Brotte L. Bär K. J. (2021). The influence of heart rate variability biofeedback on cardiac regulation and functional brain connectivity. Front. Neurosci. 15:775. 10.3389/fnins.2021.691988 34267625 PMC8275647

[B47] Schurz M. Radua J. Aichhorn M. Richlan F. Perner J. (2014). Fractionating theory of mind: a meta-analysis of functional brain imaging studies. Neurosci. Biobehav. Rev. 42, 9–34. 10.1016/j.neubiorev.2014.01.009 24486722

[B48] Sitaram R. Ros T. Stoeckel L. Haller S. Scharnowski F. Lewis-Peacock J.. (2017). Closed-loop brain training: the science of neurofeedback. Nat. Rev. Neurosci. 18, 86–100. 10.1038/nrn.2016.164 28003656

[B49] Specht K. Wigglesworth P. (2018). The functional and structural asymmetries of the superior temporal sulcus. Scand. J. Psychol. 59, 74–82. 10.1111/sjop.12410 29356006

[B50] Spielberger C. D. Gorsuch R. L. Lushene R. Vagg P. R. Jacobs G. A. (1983). Manual for the State-Trait Anxiety Inventory. Palo Alto, CA: Consulting Psychologists Press.

[B51] Szameitat A. J. Shen S. Sterr A. (2007). Motor imagery of complex everyday movements. an fMRI study. Neuroimage 34, 702–713. 10.1016/j.neuroimage.2006.09.033 17112742

[B52] To W. T. de Ridder D. Hart J. Jr. Vanneste S. (2018). Changing brain networks through non-invasive neuromodulation. Front. Hum. Neurosci. 12:128. 10.3389/fnhum.2018.00128 29706876 PMC5908883

[B53] Todorov A. (2012). The role of the amygdala in face perception and evaluation. Motiv. Emot. 36, 16–26. 10.1007/s11031-011-9238-5 22448077 PMC3294209

[B54] Tolmunen T. Lehto S. M. Heliste M. Kurl S. Kauhanen J. (2010). Alexithymia is associated with increased cardiovascular mortality in middle-aged finnish men. Psychosom. Med. 72, 187–191. 10.1097/PSY.0b013e3181c65d00 19949161

[B55] Zalesky A. Fornito A. Bullmore E. T. (2010). Network-based statistic: identifying differences in brain networks. Neuroimage 53, 1197–1207. 10.1016/j.neuroimage.2010.06.041 20600983

